# 

**Published:** 1861-09

**Authors:** 


### CONTENTS.

ORIGINAL ESSAYS AND COMMUNICATIONS.

The Amalgam Question, ........ 485
Who are Dentists? By Wm. A. Pease. ........ 492

SELECTIONS.

Working in Amalgum. ........ 503
Oxygen as an Antidote to Asphyxia from Chloroform and Ether, ........ 506
Water: its History, Characteristics, Hygienic, and Therapeutic Uses, ........ 507
Disease of the Mouth, ........ 516
Remarks on Dentistry in the Army, ........ 525
Editorial ........ 529

